# Measuring Vulnerability and Resilience in the Time of Change Using Small Area Estimation

**DOI:** 10.23889/ijpds.v8i2.2184

**Published:** 2023-09-14

**Authors:** Bethany DeSalvo

**Affiliations:** 1 United States Census Bureau, Alexandria, VA, USA

The United States Census Bureau launched a new tool for national agencies and local communities, the Community Resilience Estimates (CRE). The CRE tracks social and economic vulnerability by measuring the capacity of individuals and households to cope with the external stresses of the impacts of a disaster.

From the beginning of the pandemic, the negative effects of COVID-19 have been strongly related to certain individual and household characteristics. With access to granular microdata from the Census Bureau, the CRE maps the risk assessment of local populations down to the neighborhood level and allows national and community leaders to more efficiently respond to emergencies.

Federal statistical agencies are uniquely positioned to provide the most accurate and timely measures for an individually focused community resilience indicator. We use detailed demographic and economic data about individuals to build these estimates. Having the richest data sources, the federal government can produce estimates with the most granularity, highest statistical quality, and broadest coverage, while still protecting privacy. We do this through modeling multiple sources of data using small area estimation techniques.

This work will soon be built out to include exposure data (wind, flood, heat, fire, etc) so that federal agencies and researchers can perform experimental studies for better evidenced based policy making and evaluation.

